# Buried, not erased: palynofloras in ultra-high-pressure metamorphic rocks

**DOI:** 10.1038/s41598-025-23551-5

**Published:** 2025-10-14

**Authors:** Rodolfo Carosi, Chiara Montomoli, Salvatore Iaccarino, Davide Dana, Alberto Corno, Francesco De Cesari, Amalia Spina

**Affiliations:** 1 Department of Earth Sciences, University of Torino, Via Valperga Caluso 35, 10125 Torino, Italy; 2 Department of Physics and Geology, University of Perugia, Via Alessandro Pascoli, 06123 Perugia, Italy

**Keywords:** Evolution, Solid Earth sciences

## Abstract

Fossils are rare in metamorphic rocks due to recrystallization and deformation, especially under high-pressure metamorphic conditions. Here, we report the discovery of exceptionally preserved Paleozoic palynofloras in ten graphite-rich samples collected from the Dora-Maira Massif (Western Alps), which underwent blueschist-facies to ultra-high-pressure (UHP) metamorphism (~ 28 kbar, 520 °C). These findings challenge the assumption that fossils cannot survive such conditions, offering a new biostratigraphic tool for reconstructing the geological and tectonic history of metamorphic basement in collisional orogens worldwide.

**Supplementary Information:**

The online version contains supplementary material available at 10.1038/s41598-025-23551-5.

## Introduction

Fossils are common in sedimentary rocks but become increasingly rare in metamorphic rocks, due to elevated temperatures, recrystallization, and deformation. The minerals composing fossils are often soluble and tend to dissolve or alter during metamorphism. As a result, biomineralized fossils are typically absent under blueschist- and eclogite-facies metamorphic conditions. This represents a great limitation for reconstructing the geological history of orogenic belts where sedimentary rocks suffer high-pressure (HP) metamorphism.

Nevertheless, few fossil remnants have been documented in high-grade metamorphic rocks. Fossils have been documented within amphibolite-facies rocks in the Eastern Alps^1^. In the Western Alps, fossils have been observed in greenschist- to blueschist-facies metasedimentary sequences of the “Briançonnais Zone” ^2–4^, and in the blueschist-facies oceanic metasediments^5^. In the northern Dora-Maira Massif, poorly preserved fossils have been reported in Middle Triassic–Cretaceous metasediments affected by eclogite-facies metamorphism^6,7^. Recrystallized plant spores have been observed in Triassic rocks that experienced burial to depths of approximately 35 km and metamorphism under conditions of around 360 °C and 14 kbar^8^. Another remarkable case is the discovery of exceptionally preserved Jurassic flora in blueschist-facies concretions in New Zealand^9^. Regionally metamorphosed pelites from various areas of the Northern Apennines have yielded well-preserved middle to late Permian palynoflora^10–12^.

Palynomorphs, including organic-walled microplankton (e.g., acritarchs and chitinozoans), spores, pollen, and phytoclasts (woody fragments) are typically a few to several hundred micrometers in size and are constituted by resistant organic walls rather than minerals. These walls, composed of robust polymers such as sporopollenin, pseudochitin, or chitin, are remarkably resistant to physical and chemical degradation, even at elevated temperatures and pressures^13,14^, allowing the organism’s shape to be preserved across a broad range of temperatures and pressures. Although the exact molecular structure of these biopolymers is still uncertain, one of their most distinctive features is their capacity to undergo internal reordering of their molecular structure during burial, driven by factors such as temperature, duration, geothermal flux, and fluid geochemistry. These changes are reflected in a progressive darkening of the fossil walls, from pale yellow to black, with increasing thermal maturity^15,16^. While lithostatic pressure may slow down organic maturation, its influence becomes negligible beyond 1 kbar^17,18^. Palynomorphs range from Precambrian acritarchs to those found in recent sediments and are abundant in pelitic sediments due to their hydrodynamic properties. Their preservation during sedimentation and diagenesis typically requires anoxic-dysoxic conditions^19^. Even when thermally overmature and opaque, palynomorphs can retain their morphology, allowing for taxonomic identification using reflected light or scanning-electron microscopes^20^. Remarkable examples of palynomorph preservation include chitinozoans preserved as graphitized particles at peak temperatures of 300–500 °C ^21^; spores and acritarchs in highly deformed slates exceeding 200 °C and 1–2 kbar^22^, and Cambrian acritarchs from greenschist-facies^23^.

Successful palynological investigation in metamorphic terrains requires selective sampling, tailored processing, and appropriate observation methods. Due to poor preservation, results are often limited to taxonomic identification using open nomenclature and broad morphological characteristics, resulting in lower biostratigraphic resolution than in unmetamorphosed rocks. Here, we report the discovery of preserved Paleozoic palynofloras in ten samples from ultra-high-pressure (UHP) and high-pressure (HP) tectonic units of the Dora-Maira Massif (Western Alps). The presence of well-preserved palynoflora, which have withstood UHP conditions and complex tectonic deformation through two orogenic cycles (Variscan and Alpine), provides a novel tool to unravel the geological evolution of metamorphic basements in collisional orogens worldwide, where fossils are typically considered absent.

## Geological setting

**Fig. 1:**
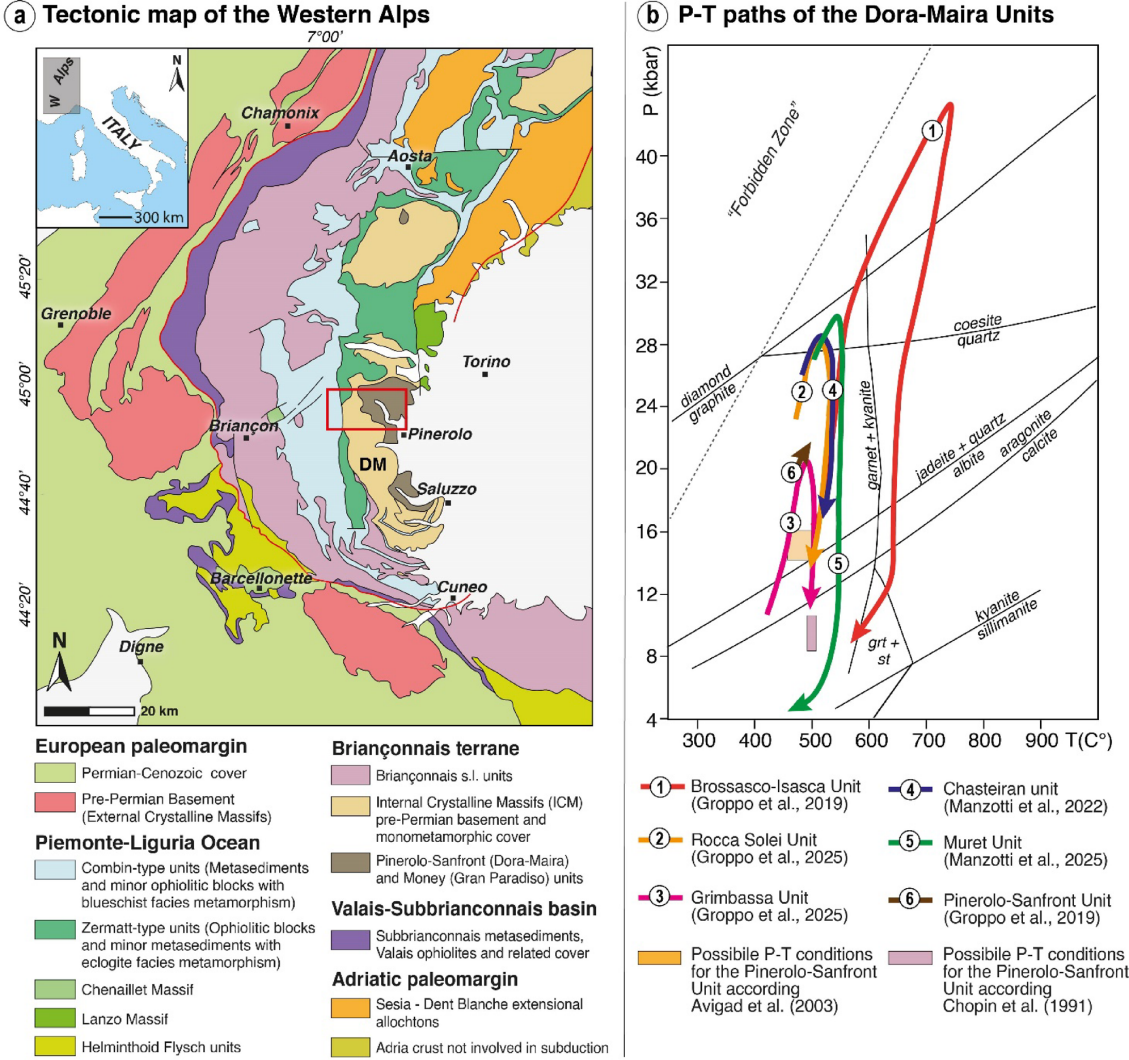
Geological setting of the study area. (a) Simplified tectonic map of the Western Alps^35^. Abbreviations: DM = Dora-Maira Massif. The red box represents the study area, reported in Fig. 2; (b) Alpine metamorphic peak P-T conditions reached by the various tectono-metamorphic units. In the case of the Pinerolo-Sanfront Unit, modern P-T estimates are not yet published in the study area. For the sake of clarity the available different estimates, made in the southern portion of the Dora-Maira Massif, are reported.

The Western Alps (Fig. 1a) formed from the collision between the Adriatic and European plates, involving paleogeographic domains that underwent different metamorphic conditions being subducted at different depths^24,25^. The Internal Crystalline Massif (Dora-Maira, Gran Paradiso, Monte Rosa), have been subducted at great depth experiencing HP metamorphism, reaching locally UHP conditions, widely described (Fig. 1b) in the Dora-Maira Massif^26–29^.

The Dora-Maira Massif consists of continental-derived units, organized in a nappe-stack made of three main tectono-metamorphic units^30,31^, which, from bottom to top, are: (a) the monocyclic (experiencing only the Alpine orogenic cycle) Pinerolo-Sanfront Unit, made of graphitic phyllite and metaconglomerate of Carboniferous age (SI Fig. S1), Permian orthogneisses, and minor Triassic quartzite; (b) the Basement Complex made of polycyclic basement and monocyclic metasedimentary and metaigneous rocks, showing Alpine peak metamorphism in eclogite-facies^28,30,33^ and (c) the Dronero Unit made of Permo-Carboniferous micaschist and Permo-Triassic metarkose.

**Fig. 2:**
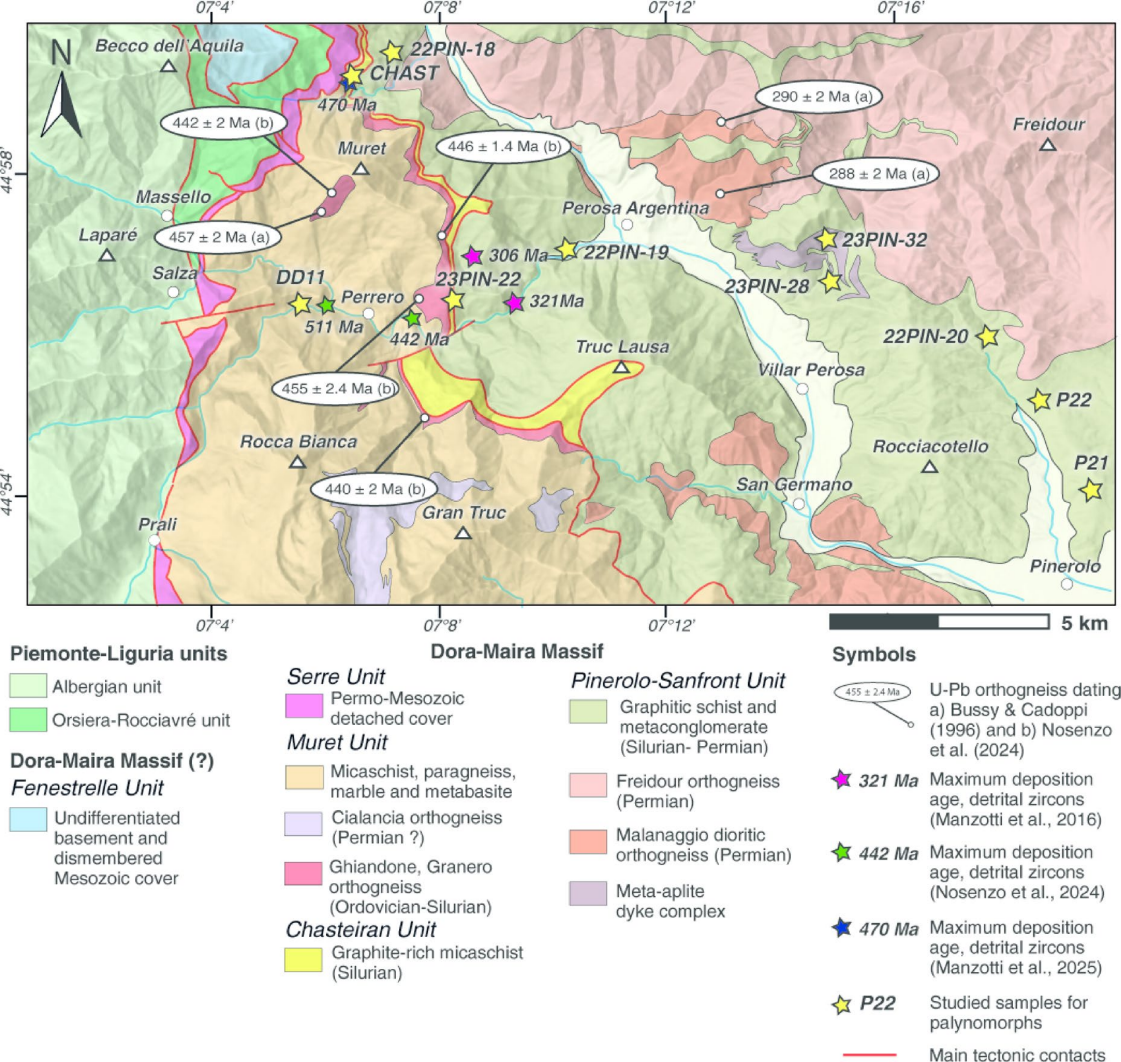
Tectonic map of the studied area, based on literature^36^ and our own original data, showing the location of studied samples (see also Table 1).

In the southern part of the massif, in the Basement Complex, several units have been identified based on different peak P-T conditions, reaching UHP conditions (Fig. 1b) in the case of the Brossasco-Isasca^26,34^, and Rocca Solei Units^29^. In the northern Dora-Maira, following the new findings of coesite^27,28^ (Fig. 1b), the “Basement Complex” has been subdivided here into three UHP units, from bottom to top: (1) the Chasteiran Unit, composed of chloritoid-rich micaschist and graphitic schist; (2) the Muret Unit, consisting of polycyclic basement rocks; and (3) the Serre Unit, comprising the Permo-Mesozoic cover (Fig. 2, SI Fig. S2).

### Study samples

Ten samples of graphitic phyllite and graphitic micaschist from three tectonic units of the northern Dora-Maira Massif (Table 1; Fig. 2, SI Fig. S1) were collected for palynological studies (see Material and Methods section).

The samples CHAST and 23PIN-22 are graphite-rich micaschist from the UHP Chasteiran Unit. Sample DD11 is a graphite-rich micaschist from the polymetamorphic UHP Muret Unit whereas samples 22PIN-18; 22PIN-19; 22PIN-20, 22PIN-28, 22PIN-32; P21; P22 are graphitic phyllite from the Pinerolo-Sanfront Unit (Table 1).

**Table 1:** Summary of the studied samples (see Fig. 2 for sample location).

Sample	Tectonic unit	Alpine peak metamorphism	Locality	Coordinates (WGS84 reference system)	Lithology	Age
22PIN-18	Pinerolo-Sanfront	Blueschist facies	Bourcet Chapel	N 44°59’32.45” E 7° 7’6.87”	Graphitic phyllite	Pennsylvanian - Cisuralian
22PIN-19*	Pinerolo-Sanfront	Blueschist facies	Masselli bridge	N 44°57’16’’ E 7°10’33’’	Graphitic phyllite	Pennsylvanian - Cisuralian
22PIN-20	Pinerolo-Sanfront	Blueschist facies	Albarea (San Pietro Val Lemina)	N 44°56’04’’ E 7°17’43’’	Graphitic phyllite	Pennsylvanian - Cisuralian
P21	Pinerolo-Sanfront	Blueschist facies	Costagrande (Pinerolo)	N 44°54’23’’ E 7°19’33’’	Graphitic phyllite	Pennsylvanian - Cisuralian
P22	Pinerolo-Sanfront	Blueschist facies	W of Cascina Pol (San Pietro Val Lemina)	N 44°55’17.58’’ E 7°18’35.7’’	Graphitic phyllite	Pennsylvanian - Cisuralian
23PIN-32	Pinerolo-Sanfront	Blueschist facies	Tagliaretto (Pinasca)	N 44°57’10’’ E 7°14’45”	Graphitic phyllite	Ludlow-Pridoli
23PIN-28	Pinerolo-Sanfront	Blueschist facies	Giborgo (Pinasca)	N 44°56’43’’ E 07°14’55”	Graphitic phyllite	Ludlow-Pridoli
CHAST	Chasteiran	UHP eclogite facies	Chasteiran village	N 44°59’23” E 7°06’27”	Graphitic level	Llandovery
23PIN-22	Chasteiran	UHP eclogite facies	Road between Granero and Villasecca	N 44°59’23” E 7°06’27”	Micaschist	Ludlow
DD11	Muret	UHP eclogite facies	Rabbioso bridge	N 44°56’24” E 7°05’49”	Graphitic paragneiss	Llandovery

## Results

**Fig. 3:**
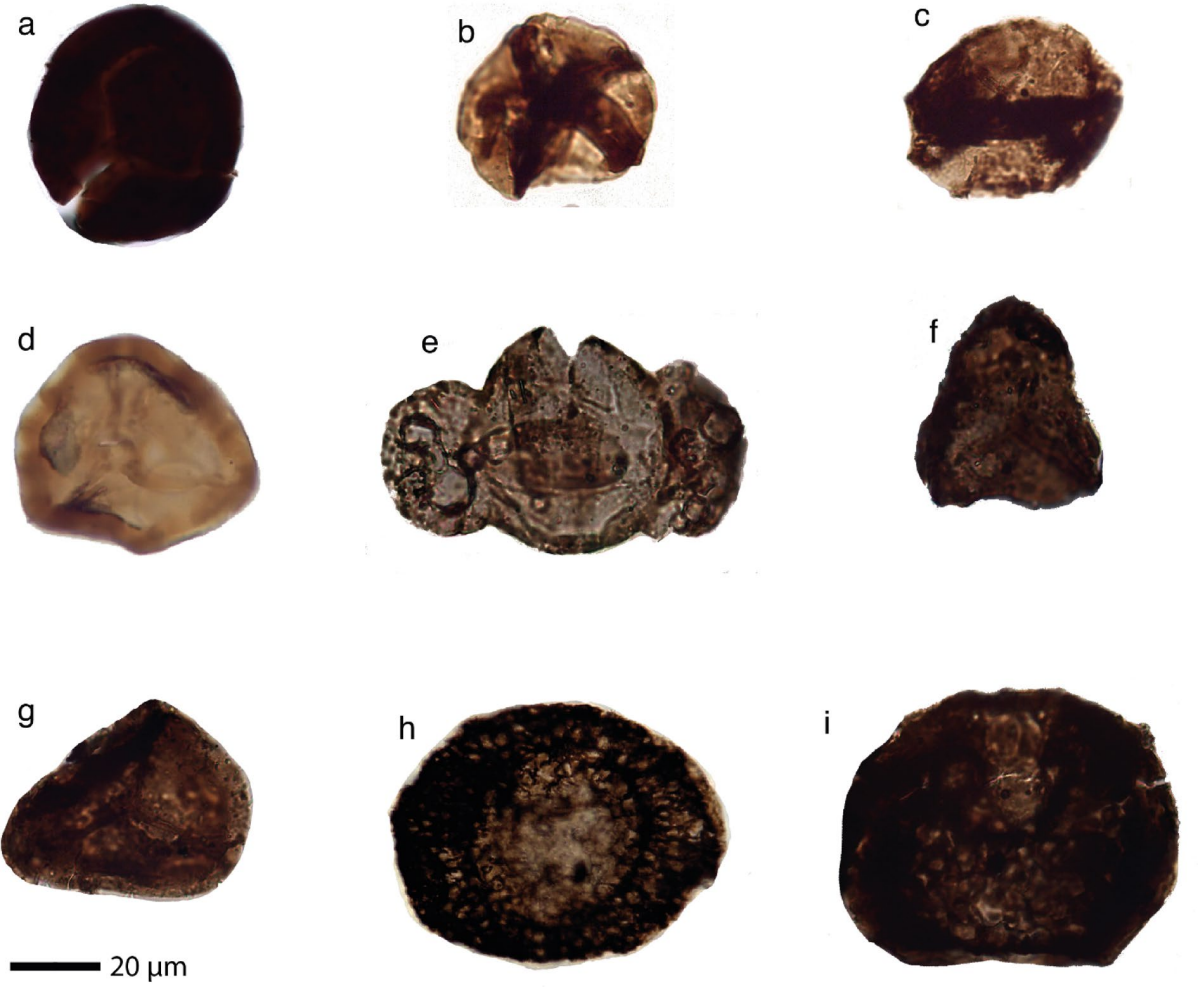
Selected sporomorphs from the studied samples (see also Table 1). a) *Ambitisporites avitus* Hoffmeister 1959 (CHAST); b). *Tetrahedraletes grayae* Strother 1991 (CHAST); c) *Dyadospora murusattenuata* (Strother and Traverse) Burgess and Richardson 1991 (CHAST); d) *Concentricosisporites* cf. *sagittarius* (Rodriguez) Richardson, Rodríguez and Sutherland 2001 (23PIN-28); e) *Hamiapollenites tractiferinus* (Samoilovich) Jansonius 1962 (22PIN-20); f) *Triquitrites* sp. (22PIN-19); g) *Lycospora* cf. *pusilla* (Ibrahim) Schopf, Wilson and Bentall 1944 (22PIN-19); h) *Florinites luberae* Samoilovich 1953 (22PIN-19); i) *Vesicaspora* sp. (22PIN-18).

We present a summary of the palynological analysis of the samples. The recovered miospore assemblages provide valuable biostratigraphic constraints on the age and paleoenvironmental interpretation of the sample. Identified taxa include cryptospores, trilete spores, and both taeniate and non-taeniate pollen grains, reflecting significant changes in terrestrial vegetation through time (Fig. 3, SI Figs. S3 and S4). Based on composition and preservation of the palynomorphs, three main assemblages were recognized: Llandovery (early Silurian), Ludlow–Pridoli (middle-late Silurian), and Pennsylvanian–Cisuralian (latest Carboniferous-early Permian).

*Samples CHAST and DD11*. Miospore assemblage mainly consists of permanent tetrad cryptospores as *Tetrahedraletes medinensis*, dyads as *Dyadospora murusattenuata* and *D. murusdensa* and monad as *Gneudnaspora divellomedia*. Smooth trilete spores as *Ambitisporites avitus*, *A. dilutus* and *Archaeozonotriletes chulus* also characterize this assemblage (Fig. 3a-c).

Age: Llandovery.

*Samples 23PIN-22; 23PIN-32; 23PIN-28.* Only few miospores as *Chelinospora poecilomorpha*, *C. cantabrica*, *Concentricosisporites* cf. *sagittarius*, *Emphanisporites rotatus* and *Retusotriletes warringtonii* were documented from these samples with such preservation to allow the characterization (Fig. 3d and SI Figs. S3 and S4).

Age: Ludlow-Pridoli.

*Samples 22PIN-18; 22PIN-19; 22PIN-20; P21; P22.* Microfloristic assemblage is mainly characterized by the presence of bisaccate non taeniate pollen grains as *Alisporites splendens*, *Alisporites* sp. in assemblage with taeniates as *Protohaploxypinus* spp. (*P. limpidus*, *P. microcorpus*) and *Hamiapollenites tractiferinus*, *H. bullaeformis*, and monosaccates as?*Potonieisporites* sp., *Florinites luberae* and *F. occultus*. Ornamented trilete spores as *Lycospora* sp. cf. *pusilla*, *Triquitrites proratus* and *Cyclogranisporites* spp. were also documented (Fig. 3e-i).

Age: latest Pennsylvanian-Cisuralian.

## Discussion

The processing methods outlined in the *Materials and Methods* section enabled the detection of abundant microfossils in ten graphite-rich rock samples collected from various tectonic units of the northern Dora-Maira Massif. These rocks underwent polyphase deformation and high- to ultra-high-pressure metamorphism (with up to 28–29 kbar of pressure) corresponding to nearly 100 km of depth into the lithosphere. This study constitutes the first report of preserved microfossils in regionally collected rocks that have experienced such extreme subduction-related metamorphic conditions. Their discovery provides a direct means of dating specific segments of the metamorphic sequence, whose ages had previously only been indirectly inferred.

### Geological implications of the inferred ages

Samples 22PIN-18, 22PIN-19, 22PIN-20, P21, and P22 from the Pinerolo–Sanfront Unit (Fig. 2) yielded palynological ages ranging from the late Pennsylvanian (late Carboniferous) to the Cisuralian (early Permian) (Table 1). These results align with previous interpretations by Novarese^37–39^, Vialon^32^ and Michard^4^, who attributed the graphite-rich metasediments and associated metaconglomerates to the Carboniferous based on lithostratigraphic criteria. Detrital zircon data^40^ further support an upper Carboniferous maximum depositional age for the metaconglomerate.

In contrast, samples 23PIN-32 and 23PIN-28, collected from the eastern portion of the Pinerolo–Sanfront Unit, yielded Silurian ages (Table 1) for the first time. Previously, it was thought that the unit consisted entirely of Carboniferous–Permian sequences. This unexpected evidence presents a significant stratigraphic or tectonic challenge. Two scenarios may explain the presence of Silurian metasediments within this unit:

- tectonic juxtaposition of Carboniferous–Permian sequences over Silurian rocks;
- an original unconformity, where Silurian deposits represent part of a polycyclic basement later overlain by younger sediments.

Given intense deformation, metamorphism, and limited outcrop exposure, field relationships remain ambiguous. However, the lack of a tectonic contact between the Silurian and Carboniferous–Permian rocks supports the second hypothesis: that the Silurian metasediments are part of a polycyclic basement sequence. These findings reveal a more complex architecture for the Pinerolo–Sanfront Unit, which includes previously unrecognized Silurian metasediments alongside Carboniferous–Permian successions.

Silurian ages were obtained from samples CHAST and 23PIN-22, both from the UHP Chasteiran Unit. Sample CHAST (early Silurian, Llandovery) is structurally positioned above the younger sample 22PIN-18 (latest Pennsylvanian–Cisuralian) from the Pinerolo–Sanfront Unit, reinforcing the interpretation of a tectonic boundary between the two units. The Silurian age obtained from CHAST and 23PIN-22 is consistent with recent detrital zircon data from Manzotti et al.^41^, who proposed a maximum depositional age of 470 Ma.

Finally, sample DD11 from the UHP Muret polycyclic unit^28^, also yielded a Silurian age. This is consistent with the occurrence of pelitic layers locally associated with black quartzite, interpreted as Silurian sediments within the original metasedimentary succession of the polycyclic basement. These rocks are lithologically comparable to well-preserved Paleozoic sequences in Sardinia and the Southern Variscan Belt^42^, reinforcing the interpretation of a widespread lower Paleozoic basement component in the Dora-Maira Massif. This data is consistent with the detrital zircon data^36^ suggesting a maximum deposition age of 500–590 Ma for DD11 host rock.

### Palynological discussions

The Silurian palynological assemblages recovered from samples CHAST, DD11, 23PIN-28, and 23PIN-32 (Table 1) provide key biostratigraphic and paleoenvironmental insights into the early Paleozoic history of the Dora-Maira Massif. These assemblages are characterized by cryptospores, particularly permanent tetrads (*Tetrahedraletes medinensis*), dyads (*Dyadospora murusattenuata*, *D. murusdensa*), and monads (*Gneudnaspora divellomedia*), alongside smooth trilete spores such as *Ambitisporites avitus*, *A. dilutus*, and *Archaeozonotriletes chulus*. This taxonomic composition is consistent with well-established Llandovery miospore assemblages reported from other regions of Gondwana and Laurussia (Laurentia, Avalonia and Baltica), supporting the reliability of the age determinations^43–49^.

Ecologically, the dominance of cryptospores and primitive trilete spores reflects a terrestrial flora composed predominantly of early land plants, such as bryophyte-grade vegetation and simple vascular plants.

The palynological evidence thus indicates that portions of the Dora-Maira basement record a phase of continental sedimentation during the Silurian, predating the Carboniferous–Permian sequences previously thought to dominate the region. These findings not only substantiate the existence of Silurian metasediments within multiple tectonic units (e.g., Chasteiran, Muret, eastern Pinerolo–Sanfront) but also support the interpretation of a polycyclic crustal architecture involving pre-Variscan components.

The ecoclimatic affinity of the land plants that produced the pollen and spores (Table 1) suggests a dry, xerophytic paleoclimatic setting. This interpretation aligns with the broader climatic transition observed in Northern Italy, from a warm and humid climate during the late Carboniferous, supported by its paleogeographic position near the equator^50^ and the presence of extensive forests dominated by ferns, horsetails, and giant lycophytes^51^, to more arid conditions at the onset of the Permian. The assembly of the supercontinent Pangaea displaced many landmasses away from the moderating influence of the oceans, contributing to a progressive trend toward climatic aridification. As a result, the lush Carboniferous forests were gradually replaced by vegetation better adapted to drier environments, such as conifers and pteridosperms. This transition from hygrophytic to xerophytic plant assemblages is also well-documented in the Permian fossil flora of Northern Italy, which reflects adaptation to increasingly arid conditions compared to the preceding period^52–55^.

### Preservation and broader implications

The exceptional preservation of palynoflora in these highly metamorphosed rocks is largely attributed to the chemical and mechanical resilience of sporopollenin, the principal component of the outer walls of spores and pollen grains. This robust biopolymer is known for its exceptional resistance to heat, pressure, and chemical alteration. Within graphite-rich matrices, sporomorphs may behave as rigid microclasts, embedded within a deformable medium. Deformation appears to be preferentially partitioned into the graphite foliation planes, sparing the sporomorphs from intense flattening and allowing them to remain “nearly rounded” in low-strain domains. In addition to the organic matter concentration techniques described in the Materials and Methods, the preservation of the recovered palynoflora may have been favored by the high metamorphic pressure, which appears to have protected sporopollenin from complete degradation and thus allowed the recognition of sporomorph content. The findings of this study underscore the transformative potential of applying palynology to metamorphic rocks. While the investigation of such altered assemblages presents significant methodological and taxonomic challenges, the ability to directly date metamorphosed rocks represents a major step forward in resolving the temporal and tectonic evolution of complex orogens. Paleopalynology in metamorphic contexts thus emerges as a powerful and sometimes underutilized tool in geological research, with the potential to revolutionize our understanding of crustal processes and basin evolution in metamorphic terranes.

## Conclusion

The results of this study provide evidence that palynomorphs can withstand even the most extreme metamorphic conditions, including ultra-high-pressure metamorphism up to 28–29 kbar (nearly 100 km of depth into the lithosphere) and intense polyphase deformation recorded during two superposed orogenic cycles. The recovery of well-preserved organic-walled palynofloras from graphite-rich metasediments of the Dora-Maira Massif, spanning from the Silurian to the Permian, demonstrates the extraordinary resilience of sporopollenin-bearing fossils, capable of survive through at least two major orogenic cycles (Variscan and Alpine).

Notably, the identification of Silurian age assemblages within the deepest tectonic unit of the Western Alps, the Pinerolo–Sanfront Unit, previously considered entirely made of Carboniferous–Permian sediment, indicates the presence of pre-Carboniferous sedimentary sequences and supports the existence of a polycyclic basement in this unit, never demonstrated before this study.

This study highlights the key role that palynological analysis can play as a complementary tool alongside geochronological, structural, and petrological approaches in reconstructing the tectonic and sedimentary evolution of crystalline basement units within collisional orogens. Future research should aim to better understand the physical and chemical conditions that allow sporopollenin and other resistant organic polymers to persist through metamorphic transformation, insights that may further enhance our ability to interpret deep-time geological processes in otherwise fossil-poor regions.

## Materials and methods

Ten samples of black phyllite and metasiltstone from the Val Germanasca - Chisone area (Table 1; Fig. 2b) were collected for palynological studies. The samples, mainly consisting of dark graphitic phyllites, were processed for palynological analyses at the Sedimentary Organic Matter Laboratory of the Department of Physics and Geology of University of Perugia (Italy). Following the standard methods^56,57^ about 30 to 100 g of each sample was cleaned with deionized water to avoid contamination, then treated by HCl37% and HF50%. The organic residue was sieved through a 10 µm filter and oxidized with Schultz solution using 99% fuming HNO₃ for ten minutes. After the Schultz treatment, a drop of the organic residue was mounted on a glass slide and examined under a stereomicroscope. If the palynomorphs appeared black, the residue was treated again with Schultz solution or chlorine or hydrogen peroxide (H₂O₂) for one hour, and the procedure was repeated as necessary. Once the organic residue yielded palynomorphs with a brown to orange color, it was filtered again through a 10 µm sieve. Palynoflora is generally bad-preserved, dark brown to black and broken. At least four slides per sample were mounted. Light microscope observations were performed on palynological slides using Leica DM1000 microscope with differential interference contrast (DIC) techniques in transmitted light. Images were captured with a Leica digital microscope camera and successively strongly corrected for contrast and brightness using GIMP opensource software.

## Supplementary Information

Below is the link to the electronic supplementary material.

Supplementary Material 1

## Data Availability

All data generated in this study are included in Supplementary material.

## Materials availability

Correspondence and requests for materials should be addressed to Rodolfo Carosi.
