# Supplementary material for: Buried, not erased: palynofloras in ultra-high-pressure metamorphic rocks
Source: Sci Rep. 2025 Oct 14;15:35865. doi: 10.1038/s41598-025-23551-5 (PMC12521529; doi:10.1038/s41598-025-23551-5)
Supplement: Supplementary file 1 — Supplementary Material 1 [file 41598_2025_23551_MOESM1_ESM.docx]

# **Supplementary information**


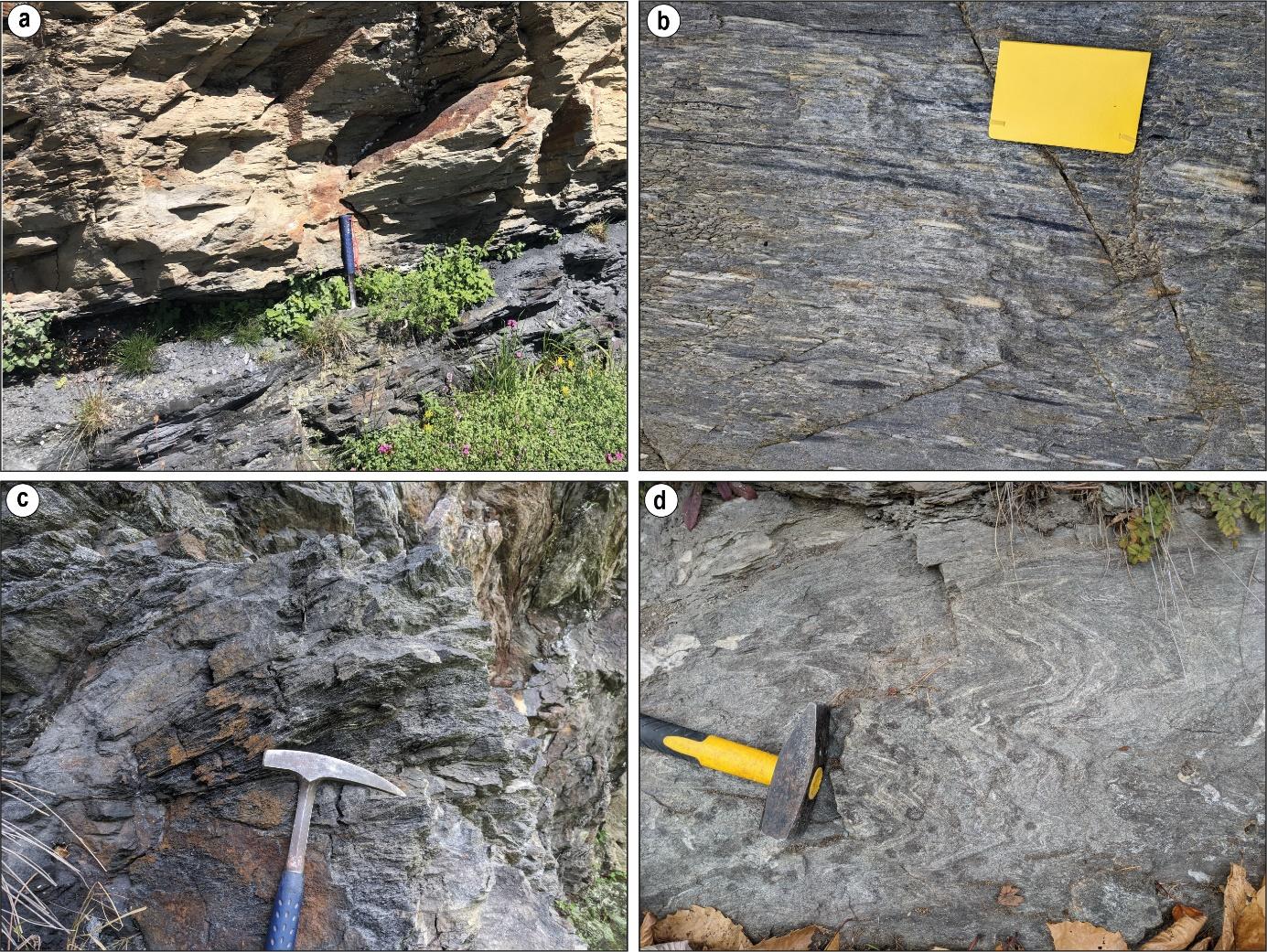


**Fig. S1.** Sampled lithologies. a) Graphitic phyllite associated to albite-bearing paragneiss of the Pinerolo-Sanfront Unit (22PIN-18); b) metaconglomerate associated to graphitic micaschist of the Pinerolo-Sanfront Unit (1 km upstream sample 22-PIN-19); c) graphitic phyllite, part of the supposed polycyclic basement hidden in the Pinerolo-Sanfront Unit (23PIN-28); d) graphitic garnet-bearing paragneiss of the Muret Unit (DD11).


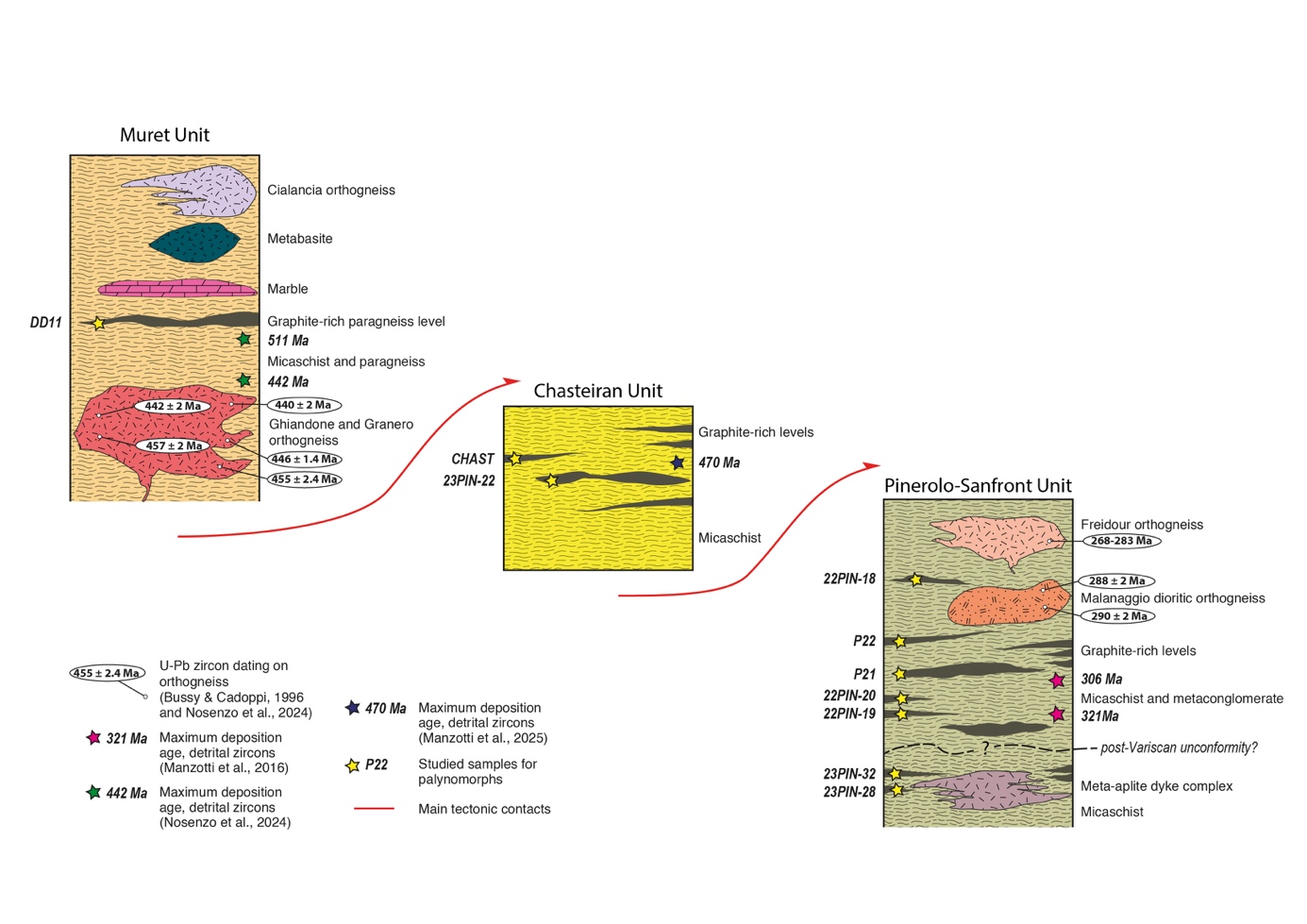


**Fig. S2**. Schematic litostratigraphic columns of the sampled tectonic units of the Northern Dora-Maira Massif.


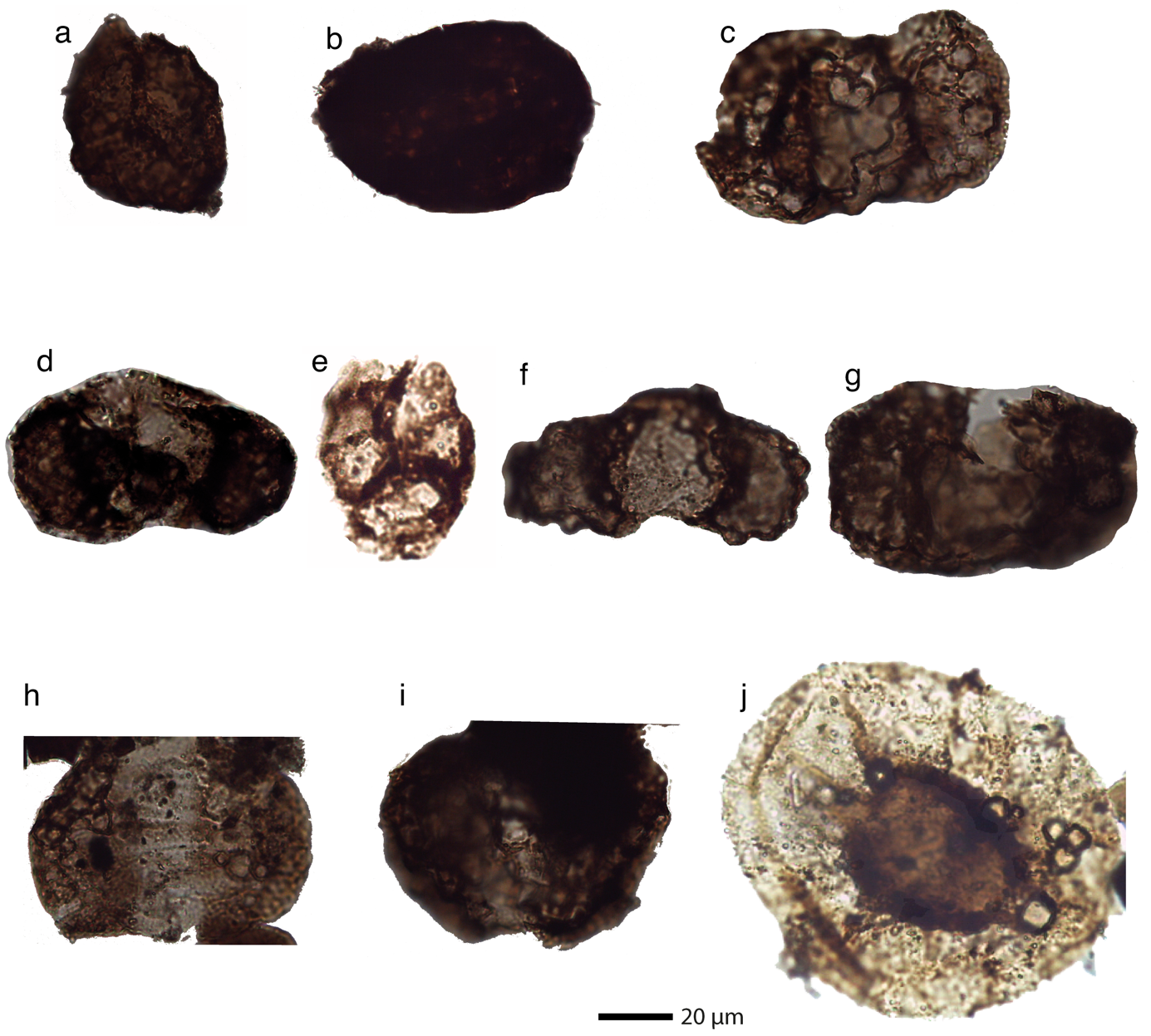


**Fig. S3***.* Selected sporomorphs from the samples studied. a) *Lycospora* sp. (22PIN18); b) *Dictyotriletes* sp. (22PIN20); c) *Alisporites australis* de Jersey 1962 (22PIN18); d) *Alisporites* sp. (22PIN19); e) *Retusotriletes* sp. (CHAST); f) *Pityosporites* sp. (22PIN20); g) *Limitisporites rectus* Leschik 1956 (22PIN18); h) *Protohaploxypinus goraiensis* (Potonié &Lele) Hart; 1964 (22PIN19); i) *Limitisporites* sp. (22PIN19); j) *Florinites occultus* Habib 1966 (22PIN19).


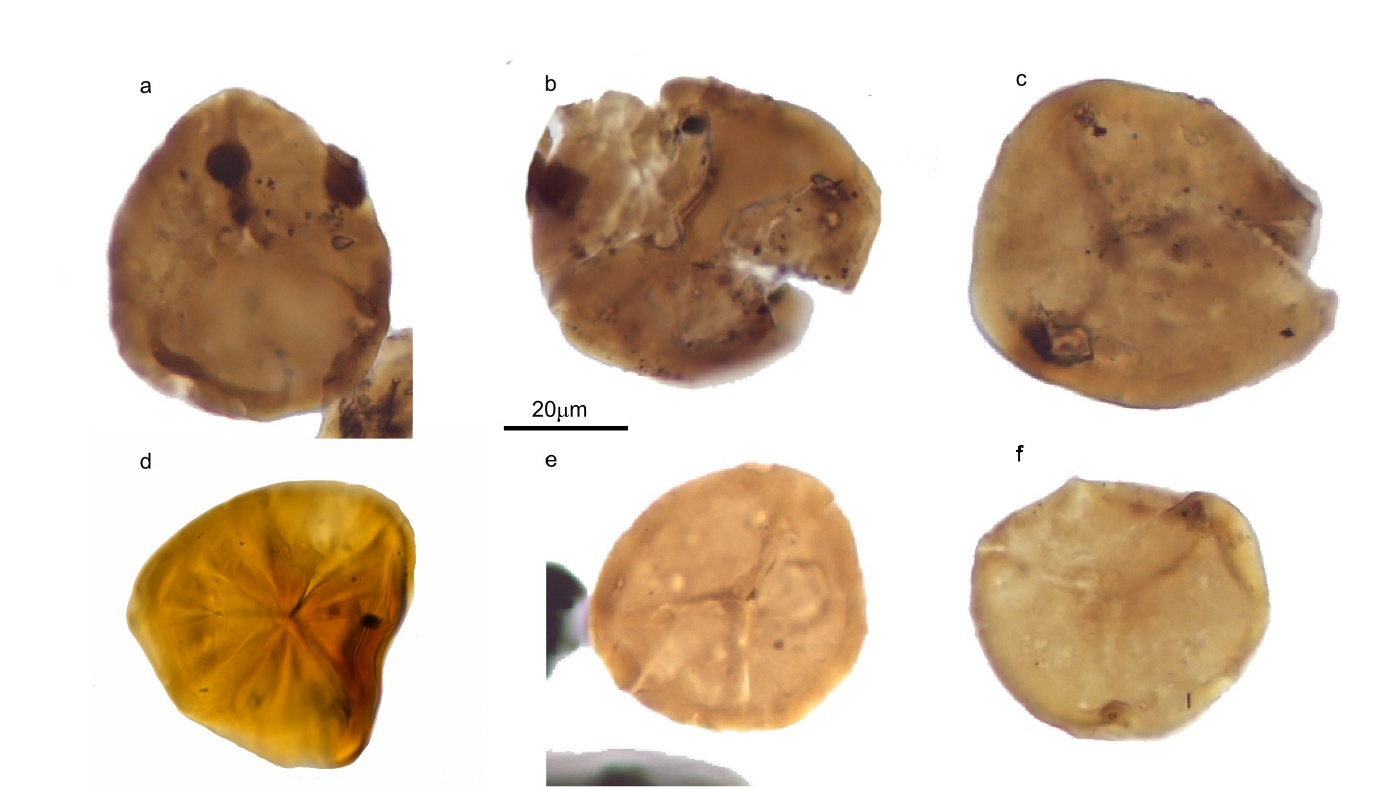


**Fig. S4**. Selected sporomorphs from the samples studied. a) *Emphanisporites* sp. (23PIN22); b) *Ambitisporites avitus* Hoffmeister 1959 (DD11); c) *Chelinospora poecilomorpha* (Richardson and Ioannides) Richardson, Rodriguez & Sutherland 2001 (23PIN22); d) *Emphanisporites rotatus* (McGregor) McGregor 1973 (23PIN22); e) *Archaeozonotriletes chulus* (Cramer) Richardson & Lister 1969 (DD11); f) *Retusotriletes warringtonii* Richardson & Lister 1969 (23PIN22).

**Samples CHAST and DD11.**

Cryptospores are dispersed spores originating from the earliest terrestrial plants, representing the first fossil evidence of non-vascular land plants. While some researchers have expanded the definition of cryptospores to include non-embryophytic terrestrial spores, such as chlorophyte spores^1^, there is general agreement regarding the embryophyte origin of these microfossils^2,3,4^. The oldest cryptospores have been identified in the Dapingian (Middle Ordovician) of northwestern Argentina ^5^. Following that, a few Darriwilian assemblages have been found in Argentina, Australia, Libya, Saudi Arabia, and Sweden, in the Gondwana and Baltica paleocontinents^6^.

By the Late Ordovician–early Silurian, cryptospores as *T. medinensis*, *D. murusattenuata*, *D. murusdensa* and *G. divellomedia* had spread globally, occurring in different paleocontinents and across various paleolatitudes. Katian cryptospore assemblages have been reported from Laurentia^7,8^, Avalonia^9, 10^, Baltica^11^, Gondwana and Peri-Gondwana^12,13^, Tarim and Siberia. For the Hirnantian period, new localities from Gondwana and Peri-Gondwana (e.g., Czechoslovakia^14^, Libya^15^, Algeria, Turkey^16^, Iran^13^, Argentina^17^, South Africa^15^), Laurentia (e.g., Anticosti Island), Baltica (Sweden^7^), Avalonia (Wales^19^) and Siberia have yielded cryptospore assemblages. Trilete spores as *Ambitisporites avitus* firstly occur in the Late Ordovician. The Ordovician records come from Turkey^16^, Algeria, Saudi Arabia^12^, Argentina^17^, Colombia^18^ and Iran^13^, all of them from Gondwana, but it colonized all the other palaeocontinents only in the Llandovery (early Silurian). In this time interval, microflora with the same morphological features of that one recorded in this study was documented worldwide, i.e., USA^,20,21,22,23,24^, Canada^25,26,27,28^, UK^19^, Norway^29,30^, Libya^31,32^; Bulgaria^33^; Saudi Arabia^34,35,36^; Brazil^37,38,39^, Paraguay^40,41^, Argentina^42,17^; South Africa^15^; Australia^43^ and China^44,45,46,47,48,49, 50,51^.

**Samples 23PIN-22; 23PIN-32; 23PIN-28.**

Trilete spores as *Chelinospora* *poecilomorpha*, *Concentricosisporites* cf. *sagittarius*, *Emphanisporites rotatus* and *Retusotriletes warringtonii* were found in the middle to late Silurian of the Old Red Sandstone Continent (ORSC) and adjacent Gondwanian regions^52^. *Chelinospora* sp. and *E. rotatus* characterize the *Synorisporites libycus-Chelinospora poecilomorpha* Assemblage Zone^52^ recognized in different ORSC regions and attributed to Gorstian-Ludfordian. Similar assemblages were also documented in the Ludlow of North Africa (Libya^53^; Algeria^54^; Tunisia^55^) and in the Pridoli of South America (Argentina^56, 57^; Brazil^58^). The other form *C. cantabrica* occurs in the *Scyalospora elegans-Iberoespora cantabrica* Spore Biozone of Spain^59^.

**Samples 22PIN-18; 22PIN-19; 22PIN20; P21; P22.**

The present microfloristic assemblage shows close similarities with latest Pennsylvanian (Gzhelian) and Cisuralian palynoflora mainly from Euramerica palaeofloristic province, although the microflora from the Cisuralian and also Guadalupian of Western Europe is generally scarce due to the presence of red beds and coarse-grained facies, which hinder good preservation^60,61,62,63,64,65,66,67,68^.

Trilete spore *Lycospora pusilla* was considered as particularly characteristic of the Euramerican coast belts of the Pennsylvanian^69^. This form in assemblage with monosaccate pollen grains as *Florinites* characterize the Moscovian-Kasimovian (late Pennsylvanian) TN miospore assemblage documented in Sardinia^70^. Other similarities of the Pinerolo assemblage were noted with the Cisuralian microflora of Southern Alps. In the Guncina Formation (Athesian District; late Kungurian), was documented a microfloristic assemblage also consisting of *Alisporites*, *Protohaploxypinus*, *Hamiapollenites*, *Potonieisporites* and *Florinites*^71,64,63,65^. A similar microflora was also documented in the Cisuralian from other successions of Central Southern Alps among the Monte Luco, Verano Collio and Tregiovo formations^72,73,74,62, 75,76,77,78^.

# **References**

1. Strother, P. K. & Beck, J. H. Spore-like microfossils from Middle Cambrian strata: expanding the meaning of the term cryptospore. In Harley, M. M., Morton, C. M., & Blackmore, S. *Pollen and spores: morphology and biology*. Royal Botanic Gardens, Kew, 413-424 (2000).
2. Steemans, P. Miospore evolution from the Ordovician to the Silurian. *Rev. Palaeobot. Palynol.* **113**, 189-196 (2000).
3. Wellman, C. H. & Gray, J. The microfossil record of early land plants. *Philos. Trans. R. Soc. Lond. B Biol. Sci.* **355**, 717-732 (2000).
4. Steemans, P. et al. Origin and radiation of the earliest vascular land plants. *Science* **324**, 353-353 (2009).
5. Rubinstein, C. V., Gerrienne, P., de la Puente, G. S., Astini, R. A. & Steemans, P. Early Middle Ordovician evidence for land plants in Argentina (eastern Gondwana). *New Phytol.* **188**, 365-369 (2010).
6. Badawy, A. S., Mehlqvist, K., Vajda, V., Ahlberg, P. & Calner, M. Late Ordovician (Katian) spores in Sweden: oldest land plant remains from Baltica. *GFF*, **136**(1), 16-21 (2014).
7. Gray, J. Land plant spores and the Ordovician-Silurian boundary. *Bull. Brit. Mus. (Nat. Hist.), Geol.* **43**, 351-358 (1988).
8. Strother, P. K. A classification schema for the cryptospores. *Palynology* **15**, 219-236 (1991).
9. Richardson, J.B. Late Ordovician and Early Silurian cryptospores and miospores from northeast Libya. *In*: A. El-Arnauti, B. Owens, B. Thusu. *Subsurface Palynostratigraphy of Northeast Libya*, Garyounis University Press, Benghazi, 89-109 (1988).
10. Edwards, D. & Richardson, J. B. Silurian and Lower Devonian plant assemblages from the Anglo‐Welsh Basin: a palaeobotanical and palynological synthesis. *Geol. J.* **39**(3‐4), 375-402 (2004).
11. Stempień-Sałek, M. Palynomorph assemblages from the Upper Ordovician in northern and central Poland. *Ann. Soc. Geol. Pol.* **81**, 21-61 (2011).
12. Wellman, C. H., Steemans, P. & Miller, M. A. Spore assemblages from Upper Ordovician and lowermost Silurian sediments recovered from the Qusaiba-1 shallow core hole, Qasim region, central Saudi Arabia. *Rev. Palaeobot. Palynol.* **212**, 111-126 (2015).
13. Ghavidel-Syooki, M. Biostratigraphy of acritarchs and chitinozoans in Ordovician strata from the Fazel Abad area, southeastern Caspian Sea, Alborz Mountains, northern Iran: stratigraphic and paleogeographic implications. *J. Sci. I. R. Iran*. **28**, 37-57 (2017).
14. Vavrdová, M. Further acritarchs and terrestrial plant remains from the Late Ordovician Hlánsá Treban (Czechoslovakia). *Čas. Mineral. Geol*. **33,** 1-10 (1988).
15. Gray J., Theron, J.H. & Boucot, A.J. Age of the Cedarberg Formation, South Africa and early land plant evolution. *Geol. Mag.* **123**, 445–454 (1986).
16. Steemans, P., Le Hérissé, A. & Bozdogan, N. Ordovician and Silurian cryptospores and miospores from southeastern Turkey. *Rev. Palaeobot. Palynol.* **93**, 35-76 (1996).
17. Rubinstein, C. V., de la Puente, G. S., Delabroye, A. & Astini, R. A. The palynological record across the Ordovician/Silurian boundary in the Cordillera Oriental, Central Andean Basin, northwestern Argentina. *Rev. Palaeobot. Palynol.* **224**, 14-25 (2016).
18. Rubinstein, C. V. et al. First Record of Cryptospores from the Late Ordovician–Early Silurian of Colombia: New Contribution to the Understanding of Plant Terrestrialization. *Ameghiniana*, **60**, 495-508 (2023).
19. Burgess, N.D. Silurian Cryptospores and miospores from the type Llandovery area, southwest Wales. *Palaeontology* **34**, 575–599 (1991).
20. Gray, J. & Boucot, A. J. Palynological evidence bearing on the Ordovician-Silurian paraconformity in Ohio. *Geol. Soc. Am. Bull.* **83**, 1299-1314 (1972).
21. Pratt, L. M., Phillips, T. L. & Dennison, J. M. Evidence of non-vascular land plants from the Early Silurian (Llandoverian) of Virginia, USA. *Rev. Palaeobot. Palynol.* **25**, 121-149 (1978).
22. Strother, P. K. & Traverse, A. Plant microfossils from Llandoverian and Wenlockian rocks of Pennsylvania. *Palynology* **3**, 1-21 (1979).
23. Miller, M. A. & Eames, L. E. Palynomorphs from the Silurian Medina Group (Lower Llandovery) of the Niagara Gorge, Lewiston, New York, USA. Palynology **6**, 221-254 (1982).
24. Beck, J. H. & Strother, P. K.. Miospores and cryptospores from the Silurian section at Allenport, Pennsylvania, USA. *J. Paleontol.* **82**, 857-883 (2008).
25. Duffield, S. L. Land-derived microfossils from the Jupiter Formation (upper Llandoverian), Anticosti Island, Quebec. *J. Paleontol.* **59**, 1005-1010 (1985).
26. Eley, B. E. & Legault, J. A. Palymorphs from the Manitoulin Formation (Early Llandovery) of southern Ontario. *Palynology* **12**, 49-63 (1988).
27. Beck, J. H. & Strother, P. K. Silurian spores and cryptospores from the Arisaig group, Nova Scotia, Canada. *Palynology* **25**, 127-177 (2001).
28. Richardson, J. G. & Ausich, W. I. Late Ordovician-Early Silurian cryptospore occurrences on Anticosti Island (Île d'Anticosti), Quebec, Canada. *Can. J. Earth Sci.* **44**, 1-7 (2007).
29. Dorning, K. J. & Aldridge, R. J. A preliminary investigation of palynological assemblages from the Early Silurian of Ringerike. *Palaeontological contributions from the University of Oslo* **278**, 105-108 (1982).
30. Smelror, M. Early Silurian acritarchs and prasinophycean algae from the Ringerike district, Oslo region (Norway). *Rev. Palaeobot. Palynol.* **52**, 137-159 (1987).
31. Hoffmeister, W. S. Lower Silurian plant spores from Libya. *Micropaleontology* **5**, 331-334 (1959).
32. Le Hérissé, A., Paris, F. & Steemans, P. Late Ordovician-earliest Silurian palynomorphs from northern Chad and correlation with contemporaneous deposits of southeastern Libya. *Bull. Geosci.* **83**, 483-504 (2013).
33. Lakova, I., Gocev, P. M. & Yanev, S. Palynostratigraphy and geological setting of the Lower Paleozoic allochthon of the Dervent Heights, SE Bulgaria. *Geol. Balk.* **22**, 71-88 (1992).
34. Steemans, P., Wellman, C. H. & Filatoff, J. Palaeophytogeographical and palaeoecological implications of a miospore assemblage of earliest Devonian (Lochkovian) age from Saudi Arabia. *Palaeogeogr. Palaeoclimatol. Palaeoecol.* **250**, 237-254 (2007).
35. Wellman, C. H., Higgs, K. T. & Steemans, P. Spore assemblages from a Silurian sequence in Borehole Hawiyah-151 from Saudi Arabia. In: S. Al-Hajri, B. Owens (Eds.), *Stratigraphic Palynology of the Palaeozoic of Saudi Arabia,* GeoArabia, Special Publications **1**, 116-133 (2000).
36. Merrell, A. M. & John, M. Significant new biostratigraphic horizons in the Qusaiba Member of the Silurian Qalibah Formation of central Saudi Arabia, and their sedimentologic expression in a sequence stratigraphic context. *GeoArabia* **10**, 49-92 (2005).
37. Gray, J., Colbath, G. K., de Faria, A., Boucot, A. J. & Rohr, D. M. Silurian-age fossils from the Paleozoic Paraná Basin, southern Brazil. *Geology* **13**, 521-525 (1985).
38. Le Hérissé, A., Melo, J. H. G., Quadros, L. P., Grahn, Y. & Steemans, P. Palynological characterization and dating of the Tianguá Formation, Serra Grande Group, northern Brazil. In: J.H.G. Melo, G.J.S. Terra (Eds.). *Correlação de Seqüências Paleozóicas Sudamericanas* **20**, 25-41 (2001).
39. Mizusaki, A. M., Melo, J. H. G., Vignol-Lelarge, M. L. & Steemans, P. Vila Maria Formation (Silurian, Paraná basin, Brazil): integrated radiometric and palynological age determinations. *Geol. Mag.* **139**, 453-463 (2002).
40. Gray, J., Boucot, A. J., Grahn, Y. & Himes, G. A new record of early Silurian land plant spores from the Parana Basin, Paraguay (Malvinokaffric Realm). *Geol. Mag.* **129**, 741-752 (1992).
41. Steemans, P. & Pereira, E. Llandovery miospore biostratigraphy and stratigraphic evolution of the Paraná Basin, Paraguay–Palaeogeographic implications. *Bull. Soc. Géol. France* **173**, 407-414 (2002).
42. Rubinstein, C. V. & Toro, B. A. Aeronian (Llandovery, Lower Silurian) palynomorphs and graptolites from the Lipeón Formation, eastern Cordillera, north-west Argentina. *Geobios* **39**, 103-111 (2006).
43. Foster, C. B. & Williams, G. E. Late Ordovician‐early Silurian age for the Mallowa salt of the Carribuddy Group, Canning Basin, Western Australia, based on occurrences of Tetrahedraletes medinensis. *Aust. J. Earth Sci.* **38**, 223-228 (1991).
44. Wang, Y. & Zhang, Y. Llandovery sporomorphs and graptolites from the Manbo formation, the Mojiang County, Yunnan, China. *Proc. R. Soc. B: Biol. Sci.* **277**, 267-275 (2010).
45. Wang, Y. & Ouyang, S. Discovery of Early Silurian spores from Fenggang, Northern Guizhou, and its palaeobotanical significance. *Acta Palaeontol Sin*. **70**, 220–243 (1997).
46. Li, J., Zhu, H. C. & Fang, Z. J. Microfossils from the Silurian Tataaiertage Formation of Kalpin, Xinjiang. *Acta Palaeontol Sin.* **36**, 136-143 (1997).
47. Yin, L. & He, S. Palynomorphs from the transitional sequences between Ordovician and Silurian of northwestern Zhejiang, South China. *Palynofloras and Palynomorphs of China*, 186–202 (2000).
48. Yi, W., Shu, O. & Chongyang, C. Early Silurian microfossil plants from the upper part of the Xiushan Formation in Guizhou Province, China and their palaeobotanical significance. *Palaeobotanist* **45**, 181-193 (1996).
49. Zhang, S. et al. (Ed.), Stratigraphy of the Tarim Basin, *Sci. Press, Bejing,* 81-102 (2001).
50. Yan, T., He, S., Yin, L., Qin, X. & Zhang, Z. Early Paleozoic palynomorphs of Zhejiang Province. *Chin. J. Stratigr*. **35**, 19-30 (2011).
51. Cai, X. et al. Classification and correlation of Silurian of Well TP2 in Shaya Rise, Tarim Basin. *Acta Petrol. Sin*. **28**, 2584-2590 (2012).
52. Richardson, J. B. & Mcgregor, D.C. Silurian and Devonian spore zones of the Old Red Sandstone continent and adjacent regions. *Bull. Geol. Surv. Canada*. **364**, 1–79 (1986)
53. Rubinstein, C. & Steemans, P. Miospore assemblages from the Silurian–Devonian boundary, in borehole A1-61, Ghadamis Basin, Libya. *Rev. Palaeobot. Palynol.* **118**(1-4), 397-421 (2002).
54. Kermandji, A. M. H. Silurian–Devonian miospores from the western and central Algeria. *Rev. micropaléontol.* **50**(1), 109-128 (2007).
55. Spina, A. & Vecoli, M. Palynostratigraphy and vegetational changes in the Siluro-Devonian of the Ghadamis Basin, North Africa. *Palaeogeogr. Palaeoclimatol. Palaeoecol*. **282**, 1-18 (2009).
56. García Muro, V. J., Rubinstein, C. V. & Steemans, P. Upper Silurian miospores from the Precordillera Basin, Argentina: biostratigraphic, palaeonvironmental and palaeogeographic implications. *Geol. Mag*. **151**(3), 472-490 (2014).
57. García Muro, V. J., Rubinstein, C. V. & Steemans, P. Upper Silurian miospores from the Precordillera Basin, Argentina: biostratigraphic, palaeonvironmental and palaeogeographic implications. *Geol. Mag*. **151**(3), 472-490 (2014).
58. Steemans, P., Rubinstein, C. & de Melo, J. H. G. Siluro-Devonian miospore biostratigraphy of the Urubu River area, western Amazon Basin, northern Brazil. *Geobios*. **41**(2), 263-282 (2008).
59. Richardson J. B., Rodriguez, R, M. & Sutherland S. J. E. Palynological zonation of Mid- Palaeozoic sequences from the Cantabrian Mountains, NW Spain: implications for inter-regional and interfacies correlation of the Ludford/Přídolí and Silurian/Devonian boundaries, and plant dispersal patterns Bull. Brit. Mus. (Nat. Hist*.), Geol.* **57,** 115-162 (2001).
60. Cassinis, G. & Doubinger, J. On the geological time of the typical Collio and Tregiovo continental beds in the Southalpine Permian (Italy), and some additional observations. *Atti Atti Tic. Sci. Ter.* **34**, 1-20. (1991).
61. Barth, S. & Mohr, B.A.R. Palynostratigraphically determined age of the Tregiovo sedimentary complex in relation to radiometric emplacement ages of the Atesina volcanic complex (Permian, Southern Alps, N Italy). *Neues Jahrb. Geol. Palontologie. Abh.* **192**, 273-292 (1994).
62. Forte, G., Kustatscher, E., Roghi, G. & Preto, N. The Permian (Kungurian, Cisuralian) palaeoenvironment and palaeoclimate of the Tregiovo Basin, Italy: Palaeobotanical, palynological and geochemical investigations. *Palaeogeogr. Palaeoclimatol. Palaeoecol.* **495** 186-204 (2018).
63. Forte, G., Vallé, F. & Kustatscher, E. Unveiling the evolution of the Kungurian (Cisuralian) flora in the paleotropics (Southern Alps, Northern Italy). *Rev. Palaeobot. Palynol.* **318** 104984 (2023).
64. Harkopf-Fröder, C. Palynology and palynofacies of a fluvio-lacustrine wet spot in the Permian Bolzano Volcanic Complex, northern Italy. 19th *International Congress on the Carboniferous and Permian.* *Cologne, July 29–August 2, 2019 Abstracts Book,* 142–143 (2019).
65. Valle, F. et al. Reconstructing Kungurian (Cisuralian, Permian) terrestrial environments within a megacaldera in the Southern Alps (N-Italy) using lithofacies analysis, palynology and stable carbon isotopes. *Riv. Ital. Paleontol. Stratigr.* **129**, 1-24 (2023).
66. Vallé, F., Morelli, C., Krainer, K., Roghi, G. & Kustatscher, E. Depositional environments and plant communities in the exceptional context of the Kungurian megacaldera of the Athesian Volcanic Group (Southern Alps, N-Italy). *Rev. Palaeobot. Palynol.* **324**, 105083 (2024).
67. Kustatscher, E., Forte, G., Branz, R., Vallé, F. & Kerp, H. A Kungurian flora from the Southern Alps (Northern Italy) yielding cuticles. *Rev. Palaeobot. Palynol.* **323**, 105067 (2024).
68. Kustatscher, E., Vallé, F., Lanthaler, B., Branz, R. & Hartkopf-Fröder, C. Nuskoisporites dulhuntyi from the Cisuralian and Lopingian of the Southern Alps: A morphological comparison between dispersed and in situ prepollen. *Rev. Palaeobot. Palynol.* **329**, 105157 (2024).
69. Stephenson, M. H. Permian palynostratigraphy: a global overview. *Geol. Soc. Lond. Spec. Publ.* **450** 321–347 (2018).
70. Pittau, P., Del Rio, M. & Funedda, A. Relationships between plant communities characterization and basin formation in the Carboniferous-Permian of Sardinia. *Boll. Soc. Geol. It.* **127**, 637-653 (2008).
71. Hartkopf-Fröder, C., Wood, G. D. & Krainer, K. Palynology of the Permian Bolzano Volcanic Complex, South-ern Alps, Italy, Part 1: Miospore preservation, quantitative spore color and quantitative fluorescence microscopy. *Proceedings of the IX International Palynological Congress, Houston, Texas, U.S.A.*, *1996.* *American Association of Stratigraphic Palynologists Foundation., 7*9–97 (2001).
72. Cassinis, G. & Doubinger, J. On the geological time of the typical Collio and Tregiovo continental beds in the Southalpine Permian (Italy), and some additional observations. *Pavia. Atti ticin. Sci. Terra.* **34**, 1–20 (1991).
73. Cassinis, G. & Doubinger, J. Artinskian and Ufimian palynomorph assemblages from the central Southern Alps, Italy, und their stratigraphic regional implications. In: Nairn A.E.M. and Korotev V. (Eds.), *Contribution to Eurasia geology International Congress on the Permian System of World, Perm, Russia*, **1,** 1–18 (1992)
74. Barth, S. & Mohr, B. A. Palynostratigraphically determined age of the Tregiovo sedimentary complex in relation to radiometric emplacement ages of the Atesina volcanic complex (Permian, Southern Alps, N Italy). *Neues Jahrb. Geol. Paläontol., Abh.* **192**, 273-292 (1994).
75. Marchetti, L. et al. Reconstruction of a late Cisuralian (Early Permian) floodplain lake environment: palaeontology and sedimentology of the Tregiovo Basin (Trentino-Alto Adige, Northern Italy). *Palaeogeogr. Palaeoclimatol. Palaeoecol.* **440**, 180–200 (2015).
76. Marchetti, L. et al. The Artinskian Warming Event: an Euramerican change in climate and the terrestrial biota during the early Permian. *Earth-Sci. Rev.* **226**, 103922 (2022).
77. Spina, A. et al. Palynological assemblages from the 2nd tectono-sedimentary cycle of the Southern Alps: New constraints for the onset of the Alpine cycle and regional-global inferences. *Palaeogeogr. Palaeoclimatol. Palaeoecol.* **674**, 112973 (2025).
78. Spina, A., Marchetti, L., Diez, J. B., Capezzuoli, E. & Ronchi, A. New palynostratigraphic data from the Kungurian (Cisuralian) of the Athesian District (East Southern Alps). *J. Iber. Geol.* (2024).
